# Feasibility of behavioral activation group therapy in reducing depressive symptoms and improving quality of life in patients with depression: the BRAVE pilot trial

**DOI:** 10.1186/s40814-020-00596-z

**Published:** 2020-05-07

**Authors:** Alessia D’Elia, Monica Bawor, Brittany B. Dennis, Meha Bhatt, Kathryn Litke, Kathleen McCabe, Jeff Whattam, Laura Garrick, Laura O’Neill, Scott Simons, Sandra Chalmers, Brenda Key, Stefanie Goyert, Phillip Laplante, Meredith Vanstone, Feng Xie, Gordon Guyatt, Lehana Thabane, Zainab Samaan

**Affiliations:** 1 Department of Psychiatry and Behavioural Neuroscience, McMaster University, 100 West 5th Street, Hamilton, ON Canada; 2 Population Genomics Program, Chanchlani Research Centre, McMaster University, 1280 Main St. W, Hamilton, ON Canada; 3 Department of Health Research, Evidence and Impact, McMaster University, 1280 Main St. W, Hamilton, ON Canada; 4 Mood Disorders Program, St. Joseph’s Healthcare Hamilton, 100 West 5th St, Hamilton, ON Canada; 5 Department of Medicine, McMaster University, 1280 Main St. W, Hamilton, ON Canada; 6 Biostatistics Unit, Centre for Evaluation of Medicine, Hamilton, ON Canada; 7 System-Linked Research Unit, McMaster University, 1280 Main St. W, Hamilton, ON Canada; 8 Department of Anesthesia, McMaster University, 1280 Main St. W, Hamilton, ON Canada; 9 Department of Pediatrics, McMaster University, 1280 Main St. W, Hamilton, ON Canada

**Keywords:** Behavioral activation, Behavioral group therapy, Depression, Quality of life, Pilot randomized trial

## Abstract

**Background:**

Depression impacts the lives of millions of people worldwide. Behavioral activation (BA), derived from cognitive behavioral therapy, has the potential for improving depressive symptoms in patients with depression. Studies evaluating the effectiveness of BA specifically in the context of group therapy programs in a hospital setting for patients with depression are limited. In this study, we report findings from a pilot trial evaluating group BA for major depressive disorder.

**Objective:**

The objectives of this pilot trial are to assess the potential of a full trial of BA group therapy in a large-scale tertiary care setting and to provide preliminary information about possible results regarding mood symptoms and quality of life in adults with depression.

**Methods:**

Using a parallel single-cohort pragmatic pilot randomized controlled trial design, we evaluated the potential of conducting a large trial of BA effectiveness among adults with depression. Participants were randomized to the intervention (BA in addition to usual care) or control (support group in addition to usual care) groups and were assessed weekly for 18 consecutive weeks. Participants randomized to intervention underwent 28 2-h group BA therapy visits administered by trained therapists and completed assessments to examine treatment outcomes. Feasibility was measured in terms of enrollment rates (min. 20%), completion rates of study (min. 80%), and completion rates of weekly measurement scales (min. 80%). The reporting of this pilot trial is in accordance with the CONSORT extension for randomized pilot and feasibility trials.

**Results:**

We randomized 20 individuals of mean age of 48.8 years (standard deviation = 9.7) with a DSM-5 diagnosis of major depressive disorder to intervention (*n* = 10) or control (*n* = 10) groups. Based on our feasibility criteria, our recruitment rate was excellent (20/27; 74%), study completion was found to be a moderate (80% of the total participants in both arms completed the study; BA = 100%, control = 60%), and completeness of measurements on a weekly basis was adequate overall (82%; BA = 86%, control = 79%).

**Conclusions:**

The study has demonstrated the potential feasibility to perform a larger scale trial upon modifications to the control group to avoid the low rate of study completion (60%) in this group.

**Trial registration:**

ClinicalTrials NCT02045771, Registered January 22, 2014

## Background

Depression, a complex chronic disorder affecting over 350 million people globally [1], has become the second leading cause of disability worldwide [2] and is associated with increased risk of medical comorbidity, suicide, and all-cause mortality [3, 4]. Although pharmacological treatment with antidepressant medication, the most common approach to treat depression, has shown promise for improving mood in adults [5], nearly half of patients continue to show depressive symptoms over the long term [6–8]. Given the limitations of pharmacology antidepressant treatment, it is necessary to evaluate alternate and additional treatment strategies. Further, psychotropic medications as well as depression itself are known to be associated with risk of increased body weight and other metabolic changes [9], suggesting the need for treatments for depression that do not precipitate poorer physical health outcomes or are protective against metabolic changes involved in the course of depression [10].

Psychotherapy, including psychological interventions such as cognitive behavioral therapy (CBT), has been successful in the management of depression both as a single therapy or in combination with antidepressants [11], improving the overall quality of life and coping skills and producing positive long-term results [12, 13]. CBT requires, however, extensive training and resources, as well as patients’ thorough understanding of their core beliefs and behaviors.

Behavioral activation (BA), originally a component of CBT, addresses behaviors and encourages individuals to eliminate reinforcers of depressive behaviors and connect with positive reinforcers in their environment [14]. The emergence of behavioral therapy for depression has opened opportunities for the development of simplified time-efficient treatment strategies that can have lasting positive effects on depressive symptoms and quality of life.

The evidence for BA is limited in comparison with CBT; however, it has reported advantages in the form of individual therapy for adult out-patients with depression [14]. BA is reportedly just as effective in treating symptoms of depression and reducing the risk of relapse as CBT in community samples of adults with depression [12, 15, 16]. Interestingly, a study comparing BA, cognitive therapy, and anti-depressant medication in adults with depression found BA to lead to similar outcomes as anti-depressant treatment and better outcomes than cognitive therapies [17]. A systematic review identified sixteen studies investigating behavioral activation treatment and demonstrated that changes between end of study and follow-up are not significant, suggesting that the benefits of BA are retained in follow-up [13].

While BA appears helpful in treating depressive symptoms, many studies addressing BA in treating depression tend to have small sample sizes and some biased methodology [18]. A systematic review of BA treatment for older patients with depression found significant reductions in depressive symptoms but maintained that many of these studies should be considered cautiously, suggesting the need for studies with larger sample sizes and well-developed methodology [18]. Further, many of these studies did not assess the effectiveness of BA as a group intervention in a hospital setting.

Based on the available evidence, BA has the potential for success as a cost-effective treatment intervention that requires minimal guidance from clinical staff, allowing reduced wait times and increasing the number of patients that can utilize this program [13]. In this study, we report findings from a pilot trial evaluating group BA for major depressive disorder, highlight the importance of implementing such therapies to determine their effectiveness in real-life clinical settings, and discuss planned changes for the main trial. While we previously reported the acceptability of group BA therapy among patients with depression [19], this paper details results of the pilot trial.

The objectives of the BehavioRal ActiVation for reducing dEpressive symptoms and improving quality of life in patients with depression (BRAVE) pilot trial are to test the feasibility of implementing a pragmatic randomized trial to evaluate the overall efficacy of group BA in a hospital-based setting, assess participants’ satisfaction with the program, and receive feedback to modify future treatment programs. We aimed to (1) evaluate the feasibility of the study process, including recruitment rate, completion of study, group size, and data completion; (2) assess resources needed for successful completion of the study (i.e., interview rooms, computers, time investment, clinical staffing); (3) explore the change in treatment outcomes including depressive symptoms severity and quality of life between and within intervention and control groups by presenting preliminary data; and (4) provide description of participants’ scores on any of the assessments conducted during the study as well as a description of patients’ clinical and demographic characteristics.

## Methods

This trial has been registered with ClinicalTrials.gov (identifier #NCT02045771) and was approved by the Hamilton Integrated Research Ethics Board (HIREB: 14-042). The protocol for this trial is published in *Pilot and Feasibility Studies* [20]. The reporting of this pilot trial is in accordance with the CONSORT extension for randomized pilot and feasibility trials [21, 22]. See checklist in Additional file 1.

### Study setting

This single-site study took place within the Mood Disorders Program at St. Joseph’s Healthcare Hamilton, an outpatient specialized mood disorders clinic. This is a tertiary care center receiving referrals from the Greater Hamilton and surrounding area for the consultation and management of patients who have lack of response or inadequate response to treatment in the community and therefore were referred to the tertiary mood disorders clinic. Hence, the clinic often caters to patients with the most severe and complex depressive disorders.

### Recruitment of participants

**Fig. 1:**
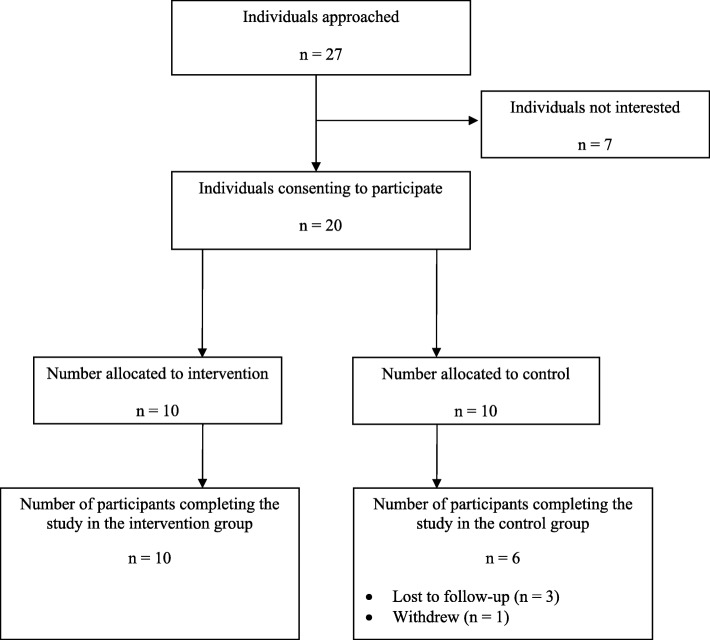
Flow diagram for participants included in the study

Clinicians approached patients at the Mood Disorders Program who were aged 18 years or older with major depressive disorders currently receiving treatment for depression at the clinic. Patients were eligible for the study if they were undergoing treatment for depression as per usual care (including antidepressants, psychotherapy, CBT, or other treatment modalities if any). Patients unable to provide written informed consent, communicate in English, or had a primary diagnosis other than depression were excluded. Details about the screening process and reasons for study incompletion were recorded (Fig. 1). Participants were allowed to discontinue participation in the study at any time. Recruitment for the pilot trial began December 2013 and ended in March 2014 when the sample size of 20 was reached. Participants were followed up at 3 months, and the pilot study ended in July 2015. Written informed consent was obtained from each participant prior to initiating any study procedures. Participants were told that the purpose of the study was to determine if the intervention is helpful and that behavioral activation was not known to be more effective than the support group. They were told that by consenting to the study, they could be randomized to receive either the intervention or the control condition and were encouraged to consult with family, friends, and clinical teams about their participation. The consent form was discussed, and the participants were given sufficient time to review the material and ask questions. Participants were provided a copy of the consent form for their own records.

### Study design

This is a pragmatic randomized controlled trial comparing group behavioral activation (group BA) in addition to usual care to support (control) group in addition to usual care. Eligible participants were randomly allocated to the intervention or control arms using a parallel group design with a 1:1 allocation ratio. A block randomization system with block sizes of 2, 4, and 6 randomly assigned allocations; the randomization schedule was computer-generated. Full details of the randomization assignment, concealment, and other trial-related methods are described in the protocol [20]. Ten participants were recruited to each arm of the study, which was decided based on the recommended therapy group size of 6–12 [20].

Following the completion of informed consent and baseline assessments including mood scales, lifestyle questionnaires, and biometric measurements, participants were randomized in blocks. A research assistant not involved in the recruitment of potential participants or the study intervention procedures allocated the participants to the trial arms using the randomization system provided and informed the participants and the therapists/study clinicians of the group allocation. All twenty participants were assigned a unique participant ID and were randomly allocated to one of the two conditions. Pieces of paper with participant IDs were mixed and drawn from an envelope, then assigned according to the randomization schedule. Following randomization, participants were asked to attend their respective groups and given a schedule for the group dates. Blinding to the intervention during treatment was not possible for participants or clinical staff. We selected names for the two groups to be similar; the intervention group was called the “Out of the Blues” group and the control group was called the “Blues Breakers” to avoid calling the groups intervention and control. The staff was then given a list of participants in their group.

### Intervention condition

The detailed methods of BA administration are described elsewhere [20]. Briefly, the intervention consisted of 28 visits across 18 weeks: twice weekly until week 10 and once weekly thereafter. Trained clinicians administered the intervention at each visit, which included study-related assessments, as described in the study protocol [20]. These clinicians were recreation therapists and social workers who provide services in the Mood Disorders clinic, trained to administer BA by completing a workshop in April 2013 and reading three BA workbooks (Michael Addis and Christopher Martell. *Overcoming Depression One Step At A time, the new behavioural activation approach to getting your life back* 2004; Jonathan Kanter, Andrew Busch and Laura Rusch. *Behavioural Activation* 2009; and Christopher Martell, Sona Dimidjian and Ruth Herman-Dunn. *Behavioural Activation for Depression, a clinician guide* 2010).

### Control condition

The control group participated in a support group for 28 sessions across 18 weeks that was led by clinicians not trained in BA. Support group for the control group was unstructured and included topics for discussions selected by the group members with a facilitator present in the room (clinician); these sessions occurred over the same period of time as that of the intervention group. A nurse trained in data collection was present for each visit and collected information pertaining to suicide risk in order to ensure patient safety, as well as answer any questions pertaining to the completion of study-related instruments.

### Data collection and instruments

An initial case report form (CRF) was designed to collect details at baseline about demographic data (i.e., age, sex, ethnicity, religious background, marital status, housing, education, employment, and income), suicidal behavior, and history of previous treatments. Physical measurements were also obtained at baseline and at the end of study using the SC-3315 Body Composition Analyzer (Tanita Corporation of America, Inc., IL, USA) for body composition data (i.e., weight, fat, muscle, bone mass, and metabolic age). Height and blood pressure were also measured.

We administered a number of instruments throughout the course of the study to monitor participants’ progress including the Beck Depression Inventory (BDI) [23], Behavioral Activation for Depression Scale (BADS) [24], Quality of Life Enjoyment and Satisfaction Questionnaire—short form (Q-LES-Q-SF) [25], Short-Form 12 Health Survey (SF-12) [26], Work and Social Adjustment Scale (WSAS) [27], Leisure Motivation Scale (LMS) [28], EuroQol 5-Dimension 5-Level (EQ-5D-5L) [29], and Response Style Questionnaire – Ruminative Response Scale (RSQ-RRS) [30]. The BDI is a tool used to measure the severity of depression that is comprised of 21 questions assigned a score between 0 and 3, with a maximum score of 63. Scores between 19 and 29 are indicative of moderate depression while those greater than 30 are associated with severe depression. The BADS is a self-administered 25-item tool used to measure activation and avoidance of activities, such as staying in bed or thinking about one’s problems, over the last 7 days, rated on a scale of “not at all” (0) to “completely” (6); higher total scores are indicative of increased activation. The Q-LES-Q-SF is a 14-item self-report instrument measuring the general quality of life (QOL) with the final score expressed as a percentage between 0 and 100%, where higher percentages are indicative of a higher QOL. WSAS is a self-report instrument measuring the level of impairment with 5 items scored between 0 (indicating no impairment) and 8 (indicating severe impairment); total scores greater than 20 indicate severe psychopathology and symptomology. The LMS is a 28-item questionnaire measuring the motivation for participating in leisure activities; this tool uses a 5-point scale for each item. The LMS generates four motivation scores: intellectual motivation, social motivation, competency or mastery motivation, and a stimulus avoidance scores, where higher scores are indicative of greater endorsement of each domain. The EQ-5D-5L questionnaire has 5 items scored from 0 to 4 and measures health state, such that higher scores are associated with poorer health. The ruminative response scale (RSS) component of the RSQ is a 22-item scale which determines an individual’s tendency to participate in ruminative coping behaviors; high scores on this scale are reflective of a high frequency of ruminative behavior.

The SF-12 is a 12-item survey to evaluate general health that generates two summary scores, the physical component score (PCS) and the mental component score (MCS); these scores range between 0 and 100, where 100 is associated with the highest level of health state. For the final question on the SF-12 instrument, an additional option of “a good bit of the time” was added. To complete scoring, reports of a “a good bit of the time” for item 12 were scored as “some of the time,” so as to not overestimate the effect of physical health on engagement in social activity.

Full details on when each data collection instrument was completed during the trial can be found in the protocol [20]. We also interviewed participants during the pilot trial using a qualitative study component to gather feedback on the study interventions. These results were reported previously [19]. Participants were followed up at 3 months post-study.

Study questionnaires and assessments were entered into a confidential electronic database (Research Electronic Data Capture, REDCap; http://project-redcap.org/). Physical forms with collected data were stored securely on-site at the Mood Disorders Program according to privacy regulations.

### Criteria for assessing trial feasibility

The following criteria were used to assess the feasibility of the current study: (1) minimum 20% recruitment rate, (2) study completion rate of 80% (i.e., 80% of data available for the final visit, consistent with other psychotherapy trials) [31–34], and (3) 80% completion of measurement instruments (i.e., the percentage of all scales completed across all participants throughout 18 weeks).

### Statistical analysis

All statistical analysis was done using R version 3.1.0 (http://www.r-project.org/) and were exploratory, therefore not intended to test the effectiveness of the intervention. Descriptive statistics are provided as mean and standard deviation (SD) or number (percent) and were used to characterize participants enrolled in the pilot study. Between-groups differences were presented as mean differences and SD. Group trajectories were plotted for each outcome to enhance visualization of group differences.

## Results

### Sample demographics

We recruited 20 individuals over a period of 4.5 months (18 weeks), with a DSM-5 diagnosis of major depressive disorder, with a mean age of 48.8 (SD = 9.7). Our sample consisted of 8 (40%) men and 12 (60%) women. Eighteen (90%) participants reported physical health issues, including medical comorbidity or symptoms (e.g., arthritis, chronic pain, hypertension, insomnia, migraines, and obesity) and 12 reported current alcohol use. Nineteen (95%) participants reported to be financially independent and receiving family and friend social support (e.g., from spouse, family, or friends). Less than half of participants have completed previous psychotherapy interventions for the treatment of depression. Six (30%) had previously received CBT, five (25%) participated in an emotion regulation skills group, four (20%) received occupational therapy, and five (25%) participated in a self-help group. Nearly half (45%) reported participating in general supportive counseling. Details of baseline demographics are described in Table 1.

**Table 1:** Baseline demographics

Characteristic	Total (***n*** = 20)	Intervention (***n*** = 10)	Control (***n*** = 10)
Men; *n* (%)	8 (40.0)	4 (40.0)	4 (40.0)
Age in years; mean (SD)	48.2 (9.6)	49.5 (9.9)	46.9 (9.6)
BMI; mean (SD)	34.4 (8.9)	35.8 (10.9)	33.1 (6.8)
Married/common law; *n* (%)	10 (50.0)	5 (50.0)	5 (50.0)
Completed post-secondary education; *n* (%)	8 (40.0)	3 (30.0)	5 (50.0)
Christian religion; *n* (%)	14 (70.0)	7 (70.0)	7 (70.0)
Have dependent children; *n* (%)	8 (40.0)	3 (30.0)	5 (50.0)
Own a house; *n* (%)	15 (75.0)	7 (70.0)	8 (80.0)
Financially independent; *n* (%)	19 (95.0)	9 (90.0)	10 (100.0)
Receiving long-term disability income; *n* (%)	8 (40.0)	3 (30.0)	5 (50.0)
Receiving social support (any)^a^; *n* (%)	19 (95.0)	9 (90.0)	10 (100.0)
Currently using alcohol; *n* (%)	12 (60.0)	6 (60.0)	6 (60.0)
History of suicide attempt; *n* (%)	3 (15.0)	2 (20.0)	1 (10.0)
Physical health issues^b^; *n* (%)	18 (90.0)	8 (80.0)	10 (100.0)
Participated in CBT; *n* (%)	6 (30.0)	2 (20.0)	4 (40.0)
Participated in emotion regulation skills group; *n* (%)	5 (25.0)	3 (30.0)	2 (20.0)
Participated in occupational therapy; *n* (%)	4 (20.0)	2 (20.0)	2 (20.0)
Participated in self-help group; *n* (%)	5 (25.0)	2 (20.0)	3 (30.0)
Participated in general supportive counseling; *n* (%)	9 (45.0)	5 (50.0)	4 (40.0)

*BMI* body mass index, *CBT* Cognitive Behavioral Therapy

^a^Social support is defined as support provided by a spouse, family members, or friends

^b^Health issues include any physical or mental comorbidity or symptoms (e.g., arthritis, chronic pain, hypertension, insomnia, migraines, and obesity)

### Feasibility results

Based on our pre-defined criteria, we assessed the feasibility of the main BRAVE trial. Of the 27 individuals approached, we successfully recruited 20 people over 4.5 months (18 weeks) to yield a recruitment rate of 74%, which fulfills our first feasibility recruitment criterion. Loss to follow-up at week 18 was moderate, with four individuals not completing the study and failing to complete the final visit; therefore, we had an overall study completion rate of 80% (second feasibility criterion). However, all four patients who dropped out were from the control group, yielding a 100% completion rate for the treatment arm and 60% for the control group. Completeness of study measurements was also adequate; intervention versus control study measurement completion rates were 85% versus 61% for the BDI, 74% versus 85% for the BADS, 87% versus 80% for the Q-LES-Q-SF, 90% versus 77% for the WSAS, 90% versus 80% for the LMS, 85% versus 80% for the EQ-5D-5L, 90% versus 80% for the RSQ-RRS instrument, and 90% versus 85% for the SF-12, respectively. The average completion rate for study instruments was 86% for the intervention group and 79% for the control group; the overall completeness of measurements for all participants throughout the study was 82%, thus fulfilling our third feasibility criterion.

Therapists providing the intervention stated that a group size between 8 and 12 participants is ideal for them to manage the group at each session. This was based on the therapists’ experience in the group setting, the size of meeting rooms available, and the time allocated for each session (2 h). The therapists also provided feedback that two clinicians are needed per group (one therapist runs the group and one therapist cofacilitates). No other resources were identified as necessary to complete the intervention in a group format.

### Intervention outcomes

**Fig. 2:**
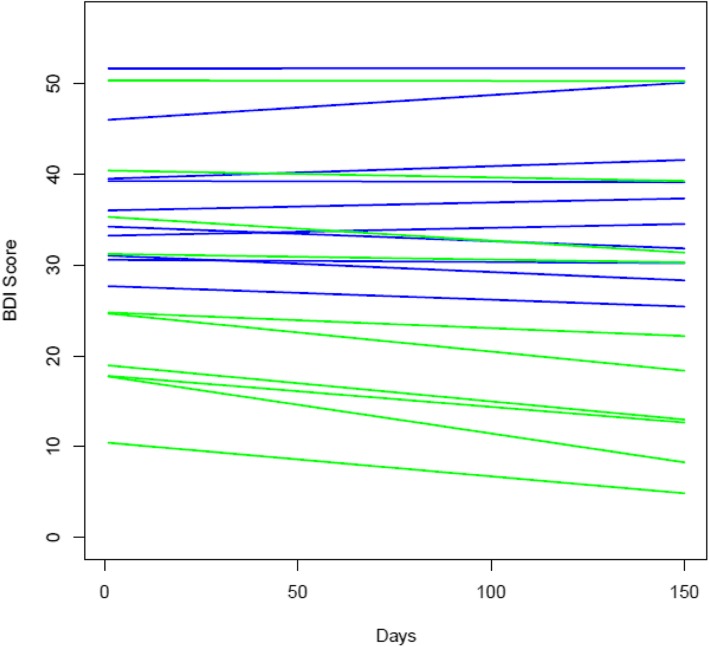
Beck Depression Inventory (BDI) scores among participant groups over the study period (*n* = 20). Note: Control = blue; BA = green. For four participants missing the final BDI scores, the last observations were carried forward in this figure

We evaluated seven study measures over the course of the 18-week study period. We provide the mean and SD of these measures for both groups at baseline and end of study (Table 2). Scores on the BDI were higher among the control group, but decreased gradually for both groups across the study period (Fig. 2). No harms were reported for either group.

**Table 2:** Mean and standard deviation (SD) for intervention and control groups at baseline and end of the pilot study

	Scores; mean (SD)	
Assessments	Baseline (screening)	End of study (week 18)
**Treatment**		
BDI	29.66 (3.29)	27.23 (3.99)
BADS	64.99 (5.92)	67.10 (7.49)
Q-LES-Q-SF	35.32 (3.66)	31.10 (4.27)
WSAS	26.37 (1.71)	29.55 (1.95)
LMS: intellectual score	39.03 (3.45)	37.77 (3.57)
LMS: social score	28.63 (3.37)	31.51 (2.67)
LMS: competency score	29.85 (3.32)	35.14 (3.05)
LMS: stimulus avoidance score	42.88 (3.01)	39.98 (3.30)
EQ-5D-5 L (health state index score)	43.19 (4.61)	43.45 (8.92)
RSQ-RRS	62.04 (3.13)	63.58 (3.39)
SF-12: PCS	33.64 (12.36)	35.17 (13.02)
SF-12: MCS	28.70 (5.94)	27.56 (10.35)
**Control**		
BDI	34.69 (3.32)	33.41 (4.10)
BADS	52.26 (7.34)	61.72 (9.62)
Q-LES-Q-SF	31.30 (3.68)	33.95 (4.60)
WSAS	30.48 (1.73)	31.44 (2.04)
LMS: intellectual score	36.67 (3.47)	38.51 (3.70)
LMS: social score	28.86 (3.39)	31.32 (2.75)
LMS: competency score	35.73 (3.36)	35.64 (3.19)
LMS: stimulus avoidance score	29.39 (3.03)	32.69 (3.50)
EQ-5D-5 L (health state index score)	50.51 (4.73)	60.51 (8.97)
RSQ-RRS	67.43 (3.32)	68.00 (3.68)
SF-12: PCS	40.35 (6.87)	36.93 (6.34)
SF-12: MCS	24.63 (7.82)	30.48 (5.79)

*BDI* Beck Depression Inventory (21 items, score range 0–63, high scores associated with greater depression); *BADS* Behavioral Activation in Depression Scale (25 items, items scored 0–6, higher score means greater activation); *Q-LES-Q-SF* Quality of Life, Enjoyment, and Satisfaction Questionnaire—Short Form (score ranges from 0–100%; maximum score associated with higher quality of life); *WSAS* Work and Social Adjustment Scale (score range 0–40, maximum score is indicative of greater impairment); *LMS* Leisure Motivation Scale (28-item, items scored 0 to 5, maximum score for each of four domains is associated with greater endorsement of each domain); *EQ-5D-5L* EuroQol 5-Dimension 5-Level (5-item, scored 0 to 5, maximum score indicating poor health state); *RSQ-RRS* Response Style Questionnaire, Ruminative Response Scale (22 items, 4-point Likert scale, high scores indicative of ruminative tendencies); *SF-12* Health Survey Short-Form 12 (score range 0–100, maximum score associated with highest health state)

### Follow-up at 3 months

We also conducted follow-up interviews with both control and intervention groups at 3 months following the study. Completion rates for follow-up interviews were 50% for both the control and treatment groups. Mean BDI scores were 30 (SD = 14.40) and 36.40 (SD = 15.45) for the intervention and control groups, respectively, thus increasing slightly for both groups compared to the final study visit.

### Anthropometry and body composition

We obtained an extensive record of participants’ physical measurements and body composition using the SC-3315 Body Composition Analyzer (Tanita Corporation of America, Inc., IL, USA) to assess changes in overall metabolic and physical health during the study (Table 3). Changes in these measures (computed as the value at the end of study minus the baseline value) were explored in order to report if any differences exist between the change observed in the intervention group versus the control group on biometric variables such as BMI, weight, and blood pressure. It is possible that BA may impact physical measures, perhaps mediated through mood or other factors; therefore, we report whether preliminary data demonstrate any difference between groups on these variables. Generally, measurements for the intervention group demonstrated positive changes; weight, waist circumference, and fat mass decreased from study baseline to week 18. Many of the measurements for the control group either increased slightly or remained constant throughout the study.

**Table 3:** Descriptive summary of participants’ physiology and body composition

	Intervention		Control	
Assessment, mean (standard deviation)	Baseline (week 1)	End of study (week 18)	Baseline (week 1)	End of study (week 18)
Height, cm (SD)	168.0 (9.8)	168.9 (9.8)	172.2 (12.3)	172.2 (12.3)
Weight, kg (SD)	93.1 (19.6)	90.4 (16.2)	100.7 (30.0)	100.4 (32.0)
BMI, kg/m^2^ (SD)	33.8 (10.9)	33.6 (7.5)	33.6 (6.8)	32.8 (5.5)
Waist circumference, cm (SD)	105.7 (12.8)	102.7 (11.9)	107.8 (19.3)	111.9 (13.4)
Hip circumference, cm (SD)	115.1 (13.0)	115.6 (14.9)	111.7 (19.0)	115.6 (14.2)
Blood pressure, systolic, mm Hg (SD)	125.7 (15.6)	126.9 (12.0)	127.9 (17.5)	132.3 (16.8)
Blood pressure, diastolic, mm Hg (SD)	77.3 (7.7)	81.9 (7.1)	81.5 (7.7)	80.4 (5.1)
Heart rate, bpm (SD)	82.5 (11.3)	82.8 (19.4)	79.3 (15.5)	77.0 (13.9)
Total fat, % (SD)	37.9 (11.9)	37.5 (11.7)	37.0 (10.6)	37.0 (8.2)
Fat mass, kg (SD)	36.2 (16.8)	34.3 (15.7)	38.7 (19.7)	37.9 (18.1)
Fat free mass, kg (SD)	56.3 (10.6)	54.3 (9.0)	61.1 (15.3)	61.1 (17.3)
Total body water, % (SD)	44. 1 (6.9)	43.7 (6.9)	45.5 (6.7)	44.9 (4.2)
Total body water mass, kg (SD)	40.2 (8.6)	38.1 (6.0)	42.0 (10.7)	44.1 (13.8)
Muscle mass, kg (SD)	53.4 (10.1)	51.5 (8.6)	58.1 (14.6)	58.1 (16.5)
Bone mass, kg (SD)	2.8 (0.5)	2.7 (0.4)	3.0 (0.7)	3.0 (0.8)
BMR, kJ (SD)	7163.4 (1320.0)	6875.6 (1011.1)	7820.4 (2011.3)	7807.9 (2280.4)
Metabolic age, years (SD)	55.6 (12.3)	57.6 (10.5)	53.5 (14.3)	56.9 (7.2)

*BMI* body mass index, *BMR* basal metabolic rate

## Discussion

The study sought to evaluate the feasibility of conducting a full randomized controlled trial to investigate the effectiveness of BA in the treatment of depression. The pilot study showed it is feasible to conduct a large BA trial based on meeting relevant feasibility criteria including recruitment rate, study completion rate, and completion of study measurement scales, though the loss to follow-up of the control group was high. This, however, is not inconsistent with other pilot trials investigating BA in the treatment of depression, which report completion rates such as 67% in the wait-list condition [33]. In order to limit the risk of loss to follow-up, participants recruited for the full trial will be compensated with parking vouchers or bus tickets. Rates of follow-up interviews at 3 months post-study were found to be low but equal between the intervention and control groups, demonstrating that further effort should be made to follow-up with participants following the end of the program. Participants will be provided the option of completing interviews over the phone or in person in order to improve adherence, accommodate participant availability, and decrease patient burden.

The BRAVE pilot trial also sought to explore the potential effectiveness of BA on depression symptoms and quality of life measures in adults with depression; preliminary data demonstrate no noticeable difference between intervention and control groups on all study measures. A full trial powered to detect clinically significant changes is needed in order to determine the effect of BA.

Behavioral activation used for the treatment of depression in a group format is practical, simple, and easy to administer; however, further research is required to understand the feasibility of this approach in a clinical setting, therefore necessitating a full trial. There are few existing trials on behavioral activation as a group therapy specifically, and further well-designed trials are needed to determine whether the use of this intervention as a therapy for patients with depression is effective in our setting or other avenues of clinical practice. This study has been designed to address these issues with proper study design and relevant methodology. RCTs effectively demonstrate differences between groups while considering relevant known confounding factors, thus making them the gold standard for clinical evidence. We had the opportunity to monitor the progress of a specific cohort of patients with depression throughout the treatment intervention and evaluate differences between groups. We were also able to observe this cohort from study initiation to completion to evaluate the feasibility of a full trial to test the effectiveness of behavioral activation in the treatment of depression.

### Key learning points

Based on our experiences with this pilot trial, we observed relatively high attrition rates, where all four individuals who did not complete the study participated in the control arm. These attrition rates are consistent with literature on psychotherapy trials, although the pattern of higher drop-out among control participants has not been previously apparent [31]. It is challenging to ascertain the true effect of this intervention relative to the control condition, as the nature of this control group is influenced by group effects, social interactions among participants, and possible attention received from group facilitators above and beyond usual care. This may indicate the need for potential modifications to the control arm of this trial in order to avoid these problems within the larger investigation. After exploring potential reasons for high attrition in the control arm, we concluded that the therapy provided in this arm of the trial (i.e., support group in addition to usual care) was not sufficient to retain participants in the study. Given these observations, it is possible that these participants are not benefitting from the study in any way and therefore lose interest over time. The time involved in conducting weekly visits and administering multiple questionnaires was considerable, and therefore may have also influenced attrition rates or completeness of assessments. We asked participants for their feedback on the pilot trial, and they reported that they wanted the intervention to be offered to all participants at the end of the study period [35]. Feedback received from participants in the control group stated that the group was not helpful for them [19]. Offering the intervention for the control group at the end of the trial may enhance motivation to complete the study period and improve the retention of the control group in the study. Given this feedback, we changed our plans for the control group for the main trial to use a wait-list group as a comparator, where participants in this group will be offered the intervention after the waiting period (approximately 18 weeks).

Participants in the intervention group were more eager to complete the study and all associated assessments, suggesting that these participants may have found it helpful in dealing with their depressive disorder, hence showing the intervention is acceptable and feasible to administer in a larger trial. This strengthens the rationale for performing the larger study, where this intervention can be explored in depth.

Unfortunately, we also observed low response rates in both groups for follow-up interviews at 3 months after study completion. An important concern in this study was loss to follow-up of the control sample where 40% of the control participants initially recruited dropped out from the study prior to completion. In the future, greater efforts should be made to maintain contact with participants following completion of the study. It may also be useful to provide the option of online completion and telephone interviews in addition to in person interviews to minimize burden to participants and encourage uptake of the follow-up. This will help to determine whether the beneficial effects of BA can be maintained following treatment completion.

The long-term goals of this program are to guide the decision-making process through evaluation of the best treatment options, with the collective efforts of primary care providers, health care specialists, and patients themselves and their families. We also intend for our study findings to be used in the development of guidelines for BA group therapy for depression.

Following the pilot trial, we have amended the study design such that participants randomized to the control condition were later given access to BA treatment. This change was made in keeping with patient feedback about the study collected through qualitative interviews post-study. Given that the study length is 18 weeks, it is possible that participants randomized to the control/wait-list condition will be lost to follow-up before the end of the wait-list period. In order to mediate this challenge, participants will be in contact with a research staff on a weekly basis for 18 weeks, during which time they will complete weekly study instruments. Weekly contact will mitigate the effect that a loss of contact may have on participant retention. No changes were made to the eligibility criteria or study intervention length, though the follow-up duration was extended. We increased the post-study follow-up to 3, 6, and 12 months in order to understand the sustainability of changes to mood and quality of life measures up until a year after the program is completed, given the chronicity of depressive disorders.

### Study strengths and limitations

This study included a comprehensive set of outcomes including depression severity, quality of life, behavioral activation, motivation, and physical health. We also collected detailed physical measurements using the Body Composition Analyzer to examine changes in body composition as an overall picture of the participants’ health.

The current pilot study design did not allow for statistical conclusions, and thus, we cannot comment on the effectiveness of the intervention based on the current pilot data; however, we were able to test the intervention feasibility. A limitation of the current pilot study is that we are unable to comment on or report whether the planned changes to the control group condition will be effective in mediating the issue of loss to follow-up and increasing retention. Furthermore, despite making use of validated instruments to assess outcomes of this intervention, these self-reported measures are at risk for recall bias as well as potentially social desirability bias.

A further limitation of the current study design is that since participants were not excluded if they were in CBT or other programs at the time of participation, and since there was no restriction for when previous programs were completed, the effects of CBT and other programs may impact mood symptoms and quality of life reported in this study. Due to the randomized design of this pilot trial, it is expected that this effect would be balanced between both groups. While a possible confounder, the purpose of this trial is to investigate the potential effectiveness of group BA in addition to care as usual; therefore, participation in co-intervention does not obscure the study objectives.

## Conclusions

This pilot study assessed the feasibility of conducting the full BRAVE randomized trial in a tertiary care mood disorders hospital-based clinic setting. We are able to conclude that based on our feasibility criteria as well as our study design, methodology, and comprehensive assessment of outcomes, the full investigation is likely to be conducted to evaluate the effectiveness of BA group therapy as a potential therapeutic approach to the treatment of major depression in adults. The caveat is the loss to follow-up of the control group. We will keep in mind the loss to follow-up challenge and have made changes in the plans regarding the selection of the comparator group. We will change the control condition to a wait-list followed by receiving the intervention and will compare the groups based on intervention versus wait-list conditions. We will also increase the post-intervention follow-up duration and have developed strategies to facilitate more successful follow-up in the main trial.

## Supplementary information

**Additional file 1.** CONSORT checklist.

## Data Availability

Raw data and materials for the study are available upon request.

## Abbreviations

CBT: Cognitive behavioral therapy
BA: Behavioral activation
BRAVE: BehavioRal ActiVation for reducing dEpressive symptoms and improving quality of life in patients with depression
HIREB: Hamilton Integrated Research Ethics Board
CRF: Case report form
BDI: Beck Depression Inventory
BADS: Behavioral Activation for Depression Scale
Q-LES-Q-SF: Quality of Life, Enjoyment, and Satisfaction Questionnaire—short form
WSAS: Work and Social Adjustment Scale
LMS: Leisure Motivation Scale
EQ-5D-5L: EuroQol 5-Dimension
RSQ-RRS: Response Style Questionnaire, Ruminative Response Scale
RedCap: Research Electronic Data Capture
REML: Restricted maximum likelihood
